# *PSRC1* May Affect Coronary Artery Disease Risk by Altering *CELSR2, PSRC1*, and *SORT1* Gene Expression and Circulating Granulin and Apolipoprotein B Protein Levels

**DOI:** 10.3389/fcvm.2022.763015

**Published:** 2022-02-18

**Authors:** Tianci Chai, Zhisheng Wang, Xiaojie Yang, Zhihuang Qiu, Liangwan Chen

**Affiliations:** ^1^Department of Cardiovascular Surgery, Fujian Medical University Union Hospital, Fuzhou, China; ^2^Key Laboratory of Cardio-Thoracic Surgery (Fujian Medical University), Fujian Province University, Fuzhou, China; ^3^Department of Anesthesiology, Xinyi People's Hospital, Xuzhou, China; ^4^The Affiliated Longyan First Hospital of Fujian Medical University, Longyan, China; ^5^Department of Thoracic Surgery, Fujian Medical University Union Hospital, Fuzhou, China

**Keywords:** low-density lipoprotein cholesterol, coronary artery disease, genome-wide association study, Mendelian randomization (MR), apolipoprotein B (apo B)

## Abstract

**Objective:**

The aim of the study was to identify additional factors that contributed to coronary artery disease (CAD).

**Methods:**

We conducted integrative analysis on publicly available data from genome-wide association studies and quantitative trait locus studies by employing Mendelian randomization methods to examine the associations of gene expression in liver cells and circulating protein levels with LDL-C and CAD.

**Results:**

We found that the mRNA expression levels of *CELSR2, PSRC1, SORT1, SYPL2, RHD, RHCE, ANGPTL3, ATXN7L2, DNAH11, FADS3, ST3GAL4, NYNRIN, CETP, EFCAB13*, and *SPTLC3* were significantly associated with LDL-C. The expression levels of *SORT1, PSRC1*, and *CELSR2* in liver cells were significantly associated with CAD. Higher expression levels of *SORT1, PSRC1*, and *CELSR2* in the liver were significantly associated with lower circulating LDL-C levels and CAD risk. *PSRC1* variants were strongly associated with *SORT1, PSRC1*, and *CELSR2* gene expression in liver cells. Higher circulating granulin and apolipoprotein B levels, which were strongly affected by *PSRC1* variants, were significantly associated with higher LDL-C levels and CAD risk, with odds ratios of 1.15 (1.10–1.19) and 1.45 (1.21–1.74), respectively.

**Conclusion:**

This study showed that regulatory SNPs in *PSRC1* may affect CAD risk by altering *CELSR2, PSRC1*, and *SORT1* gene expression in liver cells and circulating granulins and apolipoprotein B proteins.

## Introduction

Coronary artery disease (CAD) is one of the most common cardiovascular diseases and is associated with high morbidity and mortality (1). The pathogenesis of CAD is not fully understood but is known to be a process in which lipoprotein accumulates in the arteries that supply blood to the heart. Dyslipidemia is thought to be a major factor in the development of CAD, and low-density lipoprotein cholesterol (LDL-C) has been shown to be one of the most important causal risk factors. The liver is a metabolically active tissue that is important in a number of common human diseases, including obesity, diabetes, and atherosclerosis. In terms of lipoprotein metabolism, the liver is the major site of LDL-C removal from the circulation. Altered liver expression of genes involved in lipid metabolism (2–4). Hepatic *de novo* lipogenesis influences hepatic cholesterol content as well as its effects on circulating lipid levels (5). However, the association between gene expression in liver cells and CAD has not been examined systematically.

Large-scale genome-wide association studies (GWAS) have robustly identified large numbers of risk-associated genetic variants implicated in LDL-C (6–8) and CAD (9, 10). However, the GWAS identified loci were located in complex genomic regions containing multiple genes (11). It is unclear which of these genes are functionally related to LDL-C and CAD functions. The colocalization of genetic associations for disease traits with those for intermediate molecular phenotypes, such as gene expression and metabolomics, provides powerful evidence to advance hypotheses regarding the genes and pathways through which these disease-associated variants mediate their effects (12). Quantitative trait loci (QTLs) studies have shown that genetic variants are strongly associated with liver gene expression traits (13). QTL studies for gene expression and circulating proteins can offer a route to a comprehensive molecular interpretation of GWAS findings. Therefore, integrative analysis of omics data has the potential to identify pathogenic genes for CAD.

Mendelian randomization (MR) is an analytical technique that assesses the correlation between genetic alternatives to intermediate biomarkers and subsequent diseases that are supposed to be caused by intermediate biomarkers based on the random distribution of genetic variation specific to biomarkers. The recently developed two-sample multi-instrumental MR methods based on GWAS summary data provide feasible ways to integrate omics data from independent GWAS, including QTL studies on genome-wide mRNA expression and circulating protein levels (eQTL and pQTL, respectively) (14–16). Two-sample MR methods are data integration methods that have been widely applied in the identification of intermediate molecules for disease traits and therefore are effective strategies to explicate the GWAS findings. We supposed that integration of data from large-scale GWAS and QTL studies by MR methods could identify genes associated with CAD in liver cells.

In view of the well-established causal role of LDL-C in CAD, biological molecules associated with LDL-C may be risk factors for CAD. In this study, we attempted to elucidate the biological basis of the genetic associations and identify potential risk factors for CAD. We applied several data integration methods to identify potential risk factors such as gene expression in liver cells and circulating proteins for CAD by using a combination of data from GWAS, eQTL and pQTL studies. Meanwhile, as LDL-C level was the most important risk factor for CAD, the associations between gene expression in liver cells and LDL-C were also examined. Finally, we obtained association evidence on circulating protein levels and LDL-C and CAD to identify additional factors that contributed to CAD.

## Materials and Methods

### Data Resources

This study used datasets from two LDL-C and one CAD GWAS, one eQTL study in the liver, and three pQTL studies on circulating protein levels. The two LDL-C datasets were obtained from the lipid GWAS conducted by the Global Lipids Genetics Consortium (GLGC). The first GWAS evaluated the associations between almost 2.6 million SNPs and lipid levels in 188,578 Europeans (8). The dataset containing summary results of the association between almost 2.6 million SNPs and LDL-C level was downloaded at http://csg.sph.umich.edu/abecasis/public/lipids2013/, and used in our analysis. The second LDL-C GWAS was a meta-analysis of exome-wide association studies that evaluated the association between almost 292,417 variants and LDL-C levels in 47,532 East Asians and more than 300,000 individuals primarily (84%) of European descent (http://csg.sph.umich.edu/abecasis/public/lipids2017EastAsian)(6).

We used the summary results from a large-scale CAD GWAS meta-analysis conducted by the CARDIoGRAMplusC4D consortium (10). This study enrolled ~185,000 individuals who were mainly (77%) of European ancestry. The dataset contained the summary statistics of associations between almost 9.5 million SNPs and CAD tested under the additive model in the initial GWAS (http://www.cardiogramplusc4d.org/data-downloads/).

The eQTL datasets contained the *cis*-eQTL summary level data on mRNA expression levels in liver cells from the GTEx project (17). The summary data of this eQTL study are available at https://cnsgenomics.com/software/smr/#eQTLsummarydata.

We acquired pQTL summary data from three studies that examined the associations between genome-wide SNPs and circulating protein levels in thousands of individuals. The first pQTL study tested genome-wide associations between 509,946 SNPs and circulating levels of 1,124 proteins in blood samples of 1,000 individuals from the KORA study (18). The summary data was available at http://metabolomics.helmholtz-muenchen.de/pgwas/index.php?task=download. The second pQTL study performed genome-wide testing of 10.6 million imputed autosomal variants against levels of 2,994 circulating proteins in 3,301 individuals of European descent from the INTERVAL study (19). The summary data was available at http://www.phpc.cam.ac.uk/ceu/proteins/. The third pQTL study analyzed 123 metabolites in up to 24,925 individuals (20). The 123 metabolic traits were quantified by nuclear magnetic resonance spectroscopy in blood samples. The summary data was available at http://www.computationalmedicine.fi/data#NMR_GWAS.

### SMR Analysis

The summary data–based MR (SMR) approach is a kind of two-sample multi-instrumental MR method that provides feasible ways to integrate GWAS summary data with QTL data (16). SMR facilitates the goal of integrating of summary statistics from large-scale GWAS with transcriptome-wide association data. In addition, in such MR approaches, the exposure and outcome are not necessarily measured in the same samples, and the data are summary statistics rather than the raw data of the individuals (16). The instrumental variables (SNPs that were both tested in the independent QTL studies and GWAS) provided the summary data (e.g., the regression coefficient beta values and standard error) on the effects of SNPs on the levels of biomolecules and outcomes. By analyzing these summary data SMR estimates the causal effects of the biomolecules on the outcomes.

SMR software (version 0.712) is a command-line program that was downloaded from http://cnsgenomics.com/software/smr/. The parameters were left at the default setting in the analysis. The outcome data (i.e., SNP rs number, alleles, allele frequency, beta values, standard error, *P*-values) required for SMR analysis were collected from the LDL-C and CAD GWAS datasets and then organized into a.ma file with 8 columns specific for the SMR analysis by using the R program. The files containing eQTL summary data in binary format for the SMR analysis were available at http://cnsgenomics.com/software/smr/#DataResource (described above). We used genotype data of HapMap r23 CEU as a reference panel to calculate the linkage disequilibrium (LD) correlation matrix for SMR. The genome-wide significance threshold for the SMR analysis was 5.0 × 10^−6^. We also performed the heterogeneity in dependent instruments (HEIDI) test to test the “no horizontal pleiotropy” assumption. The HEIDI test was conducted to examine whether there is a single causal SNP affecting gene expression and the phenotype. Genes with *P*_HEIDI_ ≥ 0.05 (without heterogeneity) were considered. Functional annotation enrichment analyses were performed for the identified genes by using the DAVID online tools. Protein-protein interaction analyses were performed in the STRING (https://string-db.org/) and LENS (severus.dbmi.pitt.edu/LENS/) databases.

### MR Analysis on Proteins

To obtain further supporting evidence for proteins identified in pQTL analysis, we employed the inverse-variance weighted (IVW) MR (21), MR-Egger (22), MR pleiotropy residual sum and outlier (MR-PRESSO) (23) and the Causal Analysis Using Summary Effect estimates (CAUSE) (24) methods to test for potential causal relationships between circulating protein levels and LDL-C and CAD. The inverse-variance weighted method combines the ratio estimates from each IV in a meta-analysis model (21). If the associations with circulating protein levels were to lead to horizontal pleiotropy, the intercept from MR-Egger would be expected to differ from zero (22). In such cases, we interpreted the coefficient from MR-Egger as being the more valid causal estimate. Conversely, in the absence of statistical evidence for horizontal pleiotropy from the intercept on MR-Egger, we used IVW MR analysis as it retains greater power. The IVW MR and MR-Egger analyses were performed by using the MendelianRandomization R package (25). We also detected horizontal pleiotropy and outlier-corrected causal estimation by using MR-PRESSO tests (23). The outlier test in MR-PRESSO is the procedure to test for the MR assumption of no pleiotropy. The source code and documents for MR-PRESSO are available at https://github.com/rondolab/MR-PRESSO. The default parameters were used for the MR-PRESSO analysis.

Data used in these MR analyses were the pQTL data from the three studies and LDL-C (2013 GWAS) (8) and CAD (10) GWAS data that have been described above. The data required in the MR analysis (i.e., the SNP rs number, beta values, standard errors, and the *P*-values) were extracted from each of the LDL-C and CAD GWAS and pQTL datasets. Then we used the “merge” function of the R program to transform the data into a specific file (an ordinary document with 7 columns) for each protein-trait pair. In the pQTL summary data, SNPs with a *P*-value < 1.0 × 10^−4^ were selected as potential instrumental variables, except for the NMR_GWAS pQTL study (5.0 × 10^−8^). The selection criterion was set to 1.0 × 10^−4^ for the two pQTL studies because 5.0 × 10^−8^ would lead to too few instrumental variables. We clumped SNPs (LD *r*^2^ < 0.01 within 10,000 kb) based on data from Europeans from the 1,000 Genomes project using the “clump_data” function in the R package TwoSampleMR to select independent instrumental variables. The effect allele of each SNP in the LDL-C and CAD GWAS and pQTL studies was manually checked for consistency.

For proteins that passed the three MR tests, we applied the CAUSE method to account for horizontal pleiotropic effects by pathways other than those considered in the multivariable approach. CAUSE is a Mendelian randomization accounting for correlated and uncorrelated horizontal pleiotropic effects using genome-wide summary statistics (24). Data used in the CAUSE analysis were from pQTL studies and GWAS described above. We used 1,000,000 genetic variants to estimate the nuisance parameters. Other parameters were left as their defaults in the CAUSE analysis.

## Results

### Gene Expression in Liver Cells Associated With LDL-C and CAD

**Figure 1:**
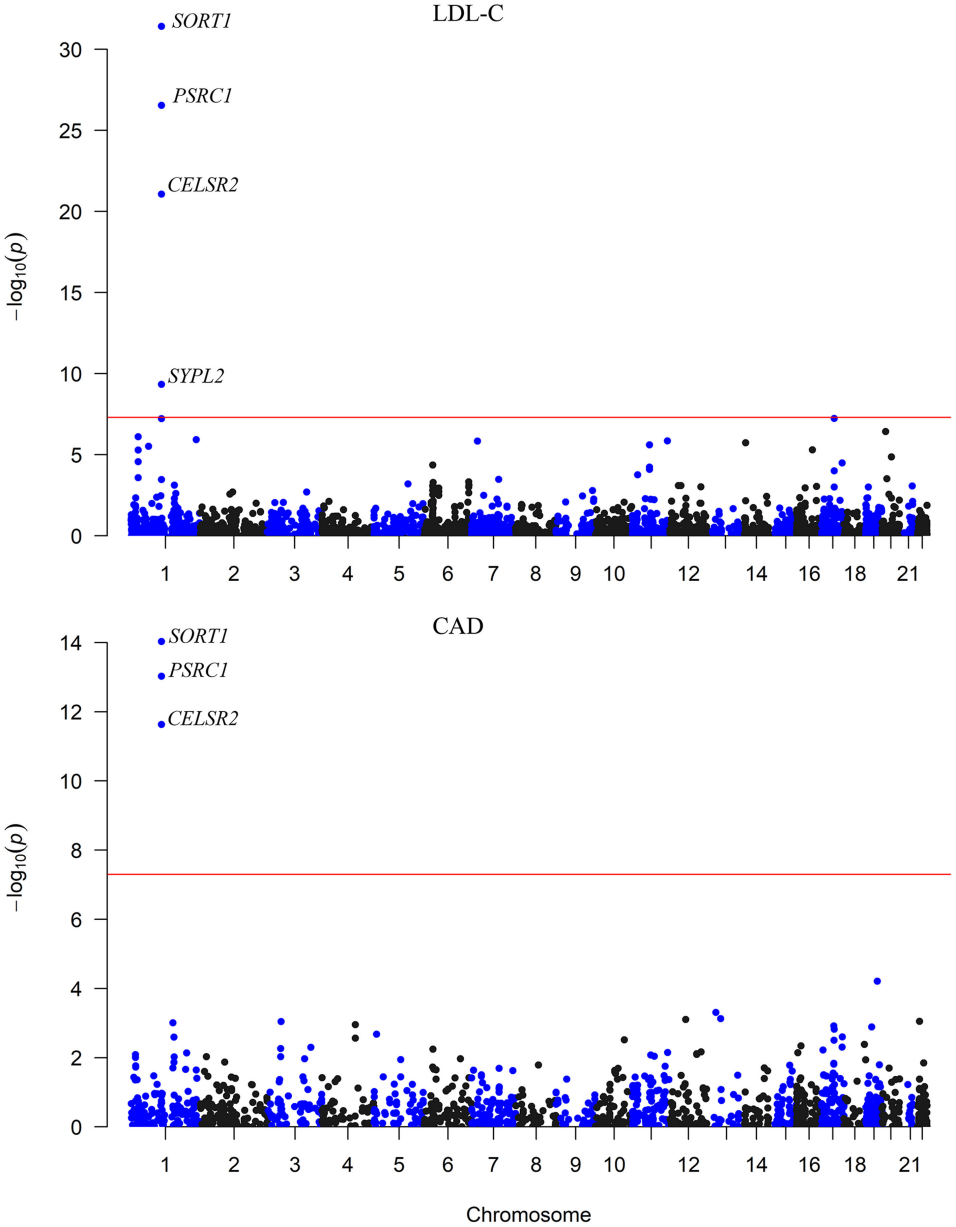
The association between mRNA expression levels and LDL-C and CAD in the liver. The x-axis represents the chromosome positions. The y axis shows the –log_10_*P*-values for the association between mRNA expression levels in liver cells and LDL-C and CAD. The associations of mRNA expression levels of *SORT1, PSRC1*, and *CELSR2* in liver cells with both LDL-C and CAD passed the significance threshold of 5.0 × 10^−8^ (red line).

We carried out an SMR analysis to find gene expression in liver cells that was associated with CAD risk by integrating eQTL data from the GTEx project with large-scale GWAS data. The expression levels of a total of 21,032 genes were analyzed, and the expression of three genes (*CELSR2, PSRC1*, and *SORT1*) was significantly associated with CAD risk (*P* < 5.0 × 10^−8^) (Figure 1). In addition, the mRNA expression of another 13 genes was nominally associated with CAD risk (Table 1; Supplementary Table S1).

**Table 1:** Liver cell gene expressions associated with CAD.

**Gene**	**CHR**	**Probe ID**	**Beta**	**SE**	***P* SMR**	***P* HEIDI**
*CELSR2*	1	ENSG00000143126.7	−0.1209	0.0172	2.35E-12	8.48E-01
*PSRC1*	1	ENSG00000134222.12	−0.0973	0.0131	9.46E-14	3.68E-02
*SORT1*	1	ENSG00000134243.7	−0.1001	0.0129	9.32E-15	3.32E-02
*CTSK*	1	ENSG00000143387.8	−0.1123	0.0341	9.82E-04	6.51E-01
*TDRD10*	1	ENSG00000163239.8	−0.0927	0.0307	2.54E-03	8.61E-01
*RBM6*	3	ENSG00000004534.10	−0.0771	0.0232	8.99E-04	4.26E-02
*SNHG18*	5	ENSG00000250786.1	−0.0482	0.0157	2.10E-03	1.88E-02
*STAT2*	12	ENSG00000170581.9	−0.0835	0.0249	7.85E-04	2.76E-02
*SNORA16*	13	ENSG00000212293.1	−0.0717	0.0206	4.95E-04	5.68E-01
*PHF11*	13	ENSG00000136147.12	0.0844	0.0250	7.41E-04	4.36E-01
*LRRC37A2*	17	ENSG00000238083.3	0.0527	0.0163	1.21E-03	2.55E-01
*GOSR2*	17	ENSG00000108433.11	0.1222	0.0385	1.51E-03	8.66E-01
*MXRA7*	17	ENSG00000182534.9	−0.0634	0.0210	2.50E-03	2.35E-02
*ZNF100*	19	ENSG00000197020.6	−0.0382	0.0119	1.30E-03	2.88E-02
*TGFB1*	19	ENSG00000105329.5	0.1456	0.0364	6.21E-05	NA
*SUSD2*	22	ENSG00000099994.10	−0.1304	0.0392	8.86E-04	7.83E-01

The associations between the expression levels of the 21,032 genes in liver cells and circulating LDL-C levels were also examined. The expression levels of 15 genes (*CELSR2, PSRC1, SORT1, SYPL2, RHD, RHCE, ANGPTL3, ATXN7L2, DNAH11, FADS3, ST3GAL4, NYNRIN, CETP, EFCAB13*, and *SPTLC3*) were significantly associated with LDL-C (*P* < 5.0 × 10^−6^) (Table 2; Supplementary Table S2). Ten of them pass the HEIDI test. These genes were enriched in gene ontology (GO) biological process of cellular lipid metabolic process (*P* = 5.0 × 10^−3^).

**Table 2:** Liver cell gene expressions associated with circulating LDL-C level.

**Gene**	**CHR**	**Probe ID**	**Beta**	**SE**	***P* SMR**	***P* HEIDI**
*RHD*	1	ENSG00000187010.14	−0.0260	0.0053	7.68E-07	6.51E-04
*RHCE*	1	ENSG00000188672.12	0.0515	0.0113	5.08E-06	8.17E-02
*ANGPTL3*	1	ENSG00000132855.4	0.1423	0.0305	3.05E-06	1.79E-01
*CELSR2*	1	ENSG00000143126.7	−0.1714	0.0179	8.39E-22	6.12E-02
*PSRC1*	1	ENSG00000134222.12	−0.1380	0.0128	2.88E-27	1.48E-01
*SORT1*	1	ENSG00000134243.7	−0.1420	0.0120	3.80E-32	9.62E-03
*SYPL2*	1	ENSG00000143028.7	−0.0335	0.0054	4.59E-10	7.39E-05
*ATXN7L2*	1	ENSG00000162650.11	−0.0510	0.0094	6.00E-08	1.58E-03
*DNAH11*	7	ENSG00000105877.13	0.0396	0.0082	1.45E-06	2.33E-01
*FADS3*	11	ENSG00000221968.4	0.1049	0.0223	2.44E-06	2.35E-01
*ST3GAL4*	11	ENSG00000110080.14	0.0870	0.0180	1.39E-06	6.85E-02
*NYNRIN*	14	ENSG00000205978.5	0.0672	0.0141	1.82E-06	1.00E-01
*CETP*	16	ENSG00000087237.6	0.1815	0.0398	4.99E-06	NA
*EFCAB13*	17	ENSG00000178852.11	0.0448	0.0083	5.83E-08	8.05E-01
*SPTLC3*	20	ENSG00000172296.8	−0.0542	0.0107	3.75E-07	3.81E-01

*CHR, Chromosome; SE, Standard error; SMR, summary data–based Mendelian randomization; HEIDI, Heterogeneity in dependent instruments*.

**Figure 2:**
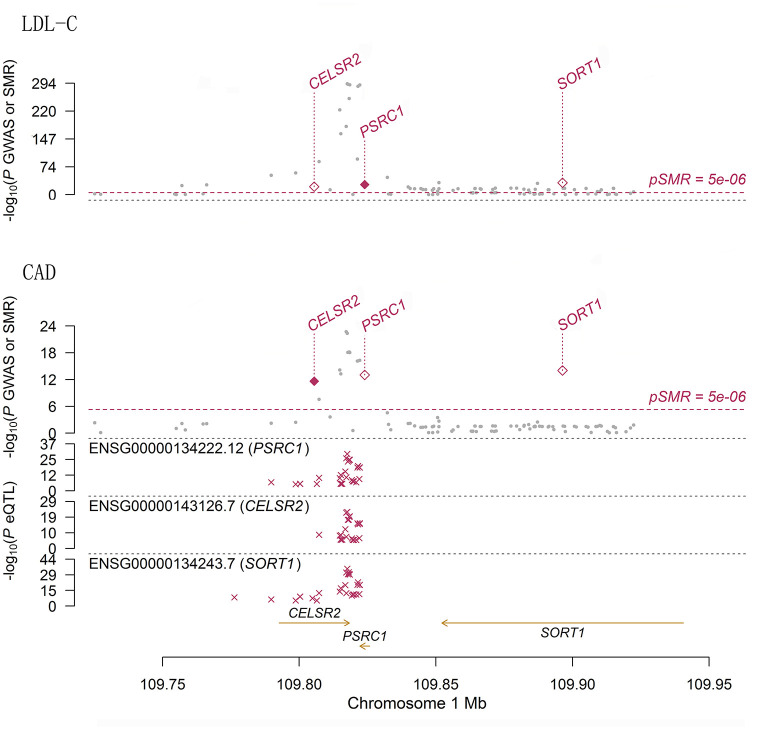
The association between mRNA expression levels of 1p13.3 genes and LDL-C and CAD in liver cells. The two panels present the results of the associations between mRNA expression levels of 1p13.3 genes and LDL-C and CAD in liver. Each panel consists of two parts. The x-axis represents the genomic position (GRCh37.p13). The lower part of each panel shows the results of eQTL. The y-axis represents –log_10_(*P* eQTL). In this part we can see that SNPs in 1p13.3 were strongly associated with the mRNA expression levels of *SORT1, PSRC1*, or *CELSR2*. The upper part of each panel shows the results of GWAS (gray dots) and SMR (red rhombus) analysis. The y-axis represents –log_10_(*P* GWAS or SMR). In this part we can see that SNPs in 1p13.3 were strongly associated with LDL-C and CAD. According to the SMR analysis, the mRNA expression levels of *SORT1, PSRC1*, and *CELSR2* in liver cells were significantly associated with LDL-C and CAD.

Therefore, the associations of the mRNA expression levels of *SORT1, PSRC1*, and *CELSR2* in the liver with both LDL-C and CAD passed the threshold of 5.0 × 10^−8^ (Figure 2). Higher expression levels of *SORT1, PSRC1*, and *CELSR2* in liver cells were associated with lower LDL-C levels (beta = −0.142, −0.138, and −0.171, respectively) and CAD risk (beta = −0.10, −0.097, and −0.121, respectively).

### pQTLs in the Identified Genes

We sought pQTLs in *SORT1, PSRC1* and *CELSR2* based on public data. In data from the KORA and INTERVAL pQTL studies, we found 116 pQTLs in *PSRC1* (Supplementary Table S3). The pQTL rs599839 was significantly associated with LDL-C (*P* = 2.75 × 10^−268^) and CAD (*P* = 5.20 × 10^−17^). This SNP was strongly associated with *SORT1* (*P* = 1.52 × 10^−56^), *PSRC1* (*P* = 2.17 × 10^−53^) and *CELSR2* (*P* = 4.73 × 10^−12^) gene expression in liver cells. In addition, this SNP was strongly associated with circulating levels of granulins in both pQTL studies (beta = −0.7453 and 0.8071, *P* = 3.32 × 10^−51^ and 1.00 × 10^−200^, respectively). Indeed, 21 SNPs in *PSRC1* were found to be associated with circulating levels of granulin. Five SNPs in *PSRC1* were strongly associated with circulating levels of apolipoprotein B.

### Proteins Causally Associated With CAD

We tested whether the two proteins, i.e., granulins and apolipoprotein B, were genetically associated with CAD using several MR methods. The results were presented in Table 3. We first examined the associations between circulating levels of these proteins and LDL-C and found that the associations between circulating levels of granulin and apolipoprotein B and LDL-C were significant in every MR analysis using data from KORA and INTERVAL studies. In datasets available from the NMR_GWAS, we only found data for apolipoprotein B among the tested metabolites. By using these data, the association between circulating levels of apolipoprotein B and LDL-C was validated.

**Table 3:** The causation between plasma protein levels and LDL-C and CAD.

**Protein levels**		**Trait/disease**	**IVW/MR-Egger**			**MR-PRESSO**		
**Study**	**Protein**		**Beta**	**SE**	***P*-value**	**Beta**	**SE**	***P*-value**
1. KORA	Granulins	LDL-C	0.262	0.019	2.95E-43	0.123	0.027	4.49E-04
2. INTERVAL	Granulins	LDL-C	0.217	0.020	1.99E-27	0.127	0.022	3.87E-05
1. KORA	Apolipoprotein B	LDL-C	0.526	0.202	9.22E-03	0.261	0.113	4.37E-02
2. INTERVAL	Apolipoprotein B	LDL-C	1.401	0.229	4.89E-10	0.676	0.123	3.89E-08
3. NMR_GWAS	Apolipoprotein B	LDL-C	1.348	0.163	1.34E-16	1.023	0.049	1.40E-09
1. KORA	Granulins	CAD	0.122	0.028	1.32E-05	0.052	0.015	1.57E-03
2. INTERVAL	Granulins	CAD	0.138	0.020	5.20E-12	0.079	0.016	4.60E-05
1. KORA	Apolipoprotein B	CAD	0.010	0.018	0.574	0.010	0.018	0.604
2. INTERVAL	Apolipoprotein B	CAD	0.289	0.105	5.92E-03	0.225	0.089	0.031
3. NMR_GWAS	Apolipoprotein B	CAD	0.373	0.093	6.05E-05	0.339	0.034	5.36E-09

*CAD, Coronary artery disease; IVW, inverse-variance weighted; LDL-C, Low-density lipoprotein cholesterol; MR, Mendelian randomization; MR-PRESSO, MR pleiotropy residual sum and outlier; SE, Standard error*.

As expected, significant associations between granulins and apolipoprotein B levels and CAD were found (Table 3). These associations were validated in several MR analyses on data from two pQTL studies. The effect of protein granulin levels on CAD risk was relatively small, with an odds ratio (OR) of ~1.15 (95% confidence interval: 1.10–1.19). The effect of protein apolipoprotein B levels on CAD risk was larger than that of granulins, with an OR of ~1.45 (95% confidence interval: 1.21–1.74). LDL-C levels were causally associated with CAD. According to the STRING database, PSRC1, APOB and GRN were connected with each other according to the STRING database (Supplementary Figure S1). Therefore, granulins, apolipoprotein B, LDL-C and CAD were genetically correlated with each other.

**Figure 3:**
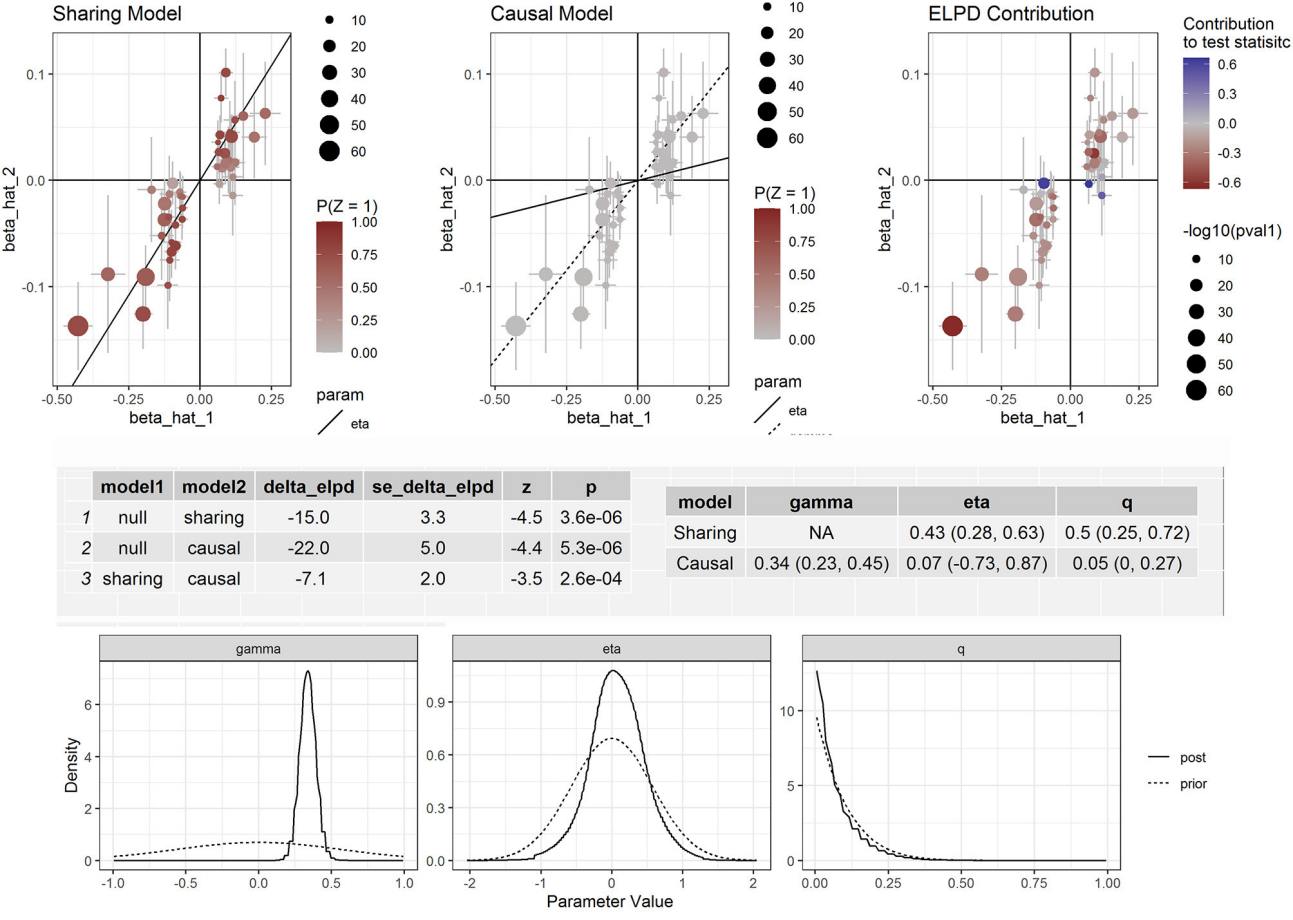
Effect-size estimates and variant-level contribution to CAUSE test statistics for circulating apolipoprotein B and CAD.

Furthermore, we corrected for correlated and uncorrelated horizontal pleiotropy using the CAUSE method and still retained an indication for the causal effect of both apolipoprotein B and LDL-C (Supplementary Figure S2) and CAD (Figure 3). By using NMR_GWAS data, we found that the causal model was significantly better than the null and sharing models for both LDL-C (*P* = 3.0 × 10^−6^ and 7.8 × 10^−3^, respectively) and CAD (*P* = 5.3 × 10^−6^ and 2.6 × 10^−4^, respectively).

## Discussion

In this study, we took advantage of MR approaches to determine potential causal factors (e.g., gene expression in liver cells and circulating protein levels) for CAD by integrating data from GWAS. We found genes that may play causal roles in LDL-C metabolism and CAD. We also found that circulating levels of granulins and apolipoprotein B were genetically associated with LDL-C and CAD. SNPs in *PSRC1* that have regulatory potential may affect CAD risk by altering *CELSR2, PSRC1*, and *SORT1* gene expression and circulating granulins and apolipoprotein B proteins.

To date, GWAS have successfully identified over 175 genetic loci for lipids. Some of these loci involved therapeutic targets such as *HMGCR* (statins), *PCSK9* (antibodies), and *NPC1L1* (ezetimibe). The causal effect of LDL-C on CAD has been well-established. Identification of factors related to LDL-C may help to understand the etiology of CAD and develop novel therapies. Elucidation of the causal factors underlying GWAS signals remains challenging due to the complexities of the genomic loci (e.g., LD) and the interactions. It is difficult to determine the most functionally relevant genes for LDL-C and CAD based only on genomic data. On the other hand, previous case-control studies have found gene expression implicated in dyslipidemia and CAD; however, these observational studies are subject to confounding and reverse causations. There was no liver gene expression profile study with large samples. MR is an analytical technique that assesses the correlation between genetic alternatives to phenotypes and outcomes based on the random distribution of genetic variation specific to biomarkers; and thus makes up for the shortcomings of traditional epidemiology studies (26, 27). By applying the two-sample MR approach, we identified the most relevant genes in the complex genomic loci for LDL-C and CAD. The findings of this study also indicated that the GWAS-identified loci contained causal factors for LDL-C and CAD, e.g., the expression of the genes in the loci.

The expression level of proline and serine rich coiled-coil 1 (*PSRC1*) in liver cells was associated with LDL-C and CAD. *PSRC1* encodes a proline-rich protein, which is a target for regulation by the tumor suppressor protein p53. The genetic associations between *PSRC1* variants and LDL-C and CAD have been well-established but the role of this gene in LDL-C and CAD has not been well-discussed. Our analysis suggested that SNPs in *PSRC1* were strongly associated with gene expression, LDL-C and CAD, and furthermore, these SNPs were significantly associated with circulating levels of granulins and apolipoprotein B. Moreover, we demonstrated that circulating levels of these proteins were causally associated with LDL-C and CAD. Circulating progranulin is a dimer of high-density lipoprotein (HDL) (28). HDL/apolipoprotein A-I is known to bind to macrophage-derived progranulin and suppress its conversion into proinflammatory granulins (29). However, how *PSRC1* interacts with *GRN* is unclear but we found that these genes were connected via other genes that were related to cardiovascular diseases or lipid metabolism (e.g., *APP, APOA1, ARFGAP1, CALR, SAMD3, LRP2, AKT2, BGN, PIK3R2, HSPG2, YY1, TGM2*) (Supplementary Figure S3). Based on multiple sources of evidence, the identified genes were suggested to be potential functional candidate genes. The interaction among these factors probably points to a pathway for LDL-C and CAD.

Our study found that apolipoprotein B was causally associated with LDL-C and CAD. It is known that one molecule of apolipoprotein B is required for the proper assembly and secretion of each very low density lipoprotein (30), which is converted into LDL-C after the hydrolysis of their triglyceride moiety (31). Indeed, apolipoprotein B is a proposed LDL-C-lowering target. Proprotein convertase subtilisin/kexin type 9 (*PCSK9*) regulates circulating LDL-C levels. Several studies have shown that PCSK9 regulates apolipoprotein B synthesis and secretion (32–34). In animals, a single dose of siRNA targeting *PCSK9* resulted in a lowering of circulating PCSK9, apolipoprotein B, and LDL-C, without measurable effects on either high density lipoprotein cholesterol or triglycerides (35). In MR analyses in human samples we showed that there was a causal effect of circulating apolipoprotein B on CAD.

This study has some limitations. First, we did not carried out basic experiment to verify the functional effect of *PSRC1* variants on gene expression and protein levels. Second, although we identified a set of causal genes, the connections between these genes were unclear. Finally, the identified “candidates” of the causal factors were inferred by statistical models. Additional studies, such as experimental studies and randomized intervention trials, are needed to strengthen the evidence. How lipid metabolism-related genes connect to each other and how they affect CAD risk need to be tested in future studies.

## Conclusion

In summary, this study elucidated the biological basis of genetic linkage and promoted the identification of susceptibility genes. The findings suggested that regulatory genetic variants in *PSRC1* may affect CAD risk through altering *CELSR2, PSRC1*, and *SORT1* gene expression in liver cells and circulating granulins and apolipoprotein B proteins, and moreover, the gene expression and circulating protein levels were genetically associated with LDL-C and CAD and may be novel risk factors for CAD. This study also provided evidence for exploring the potential causal relationship between granulins and apolipoprotein B and LDL-C and CAD. The findings may provide clues for seeking new therapeutic targets for dyslipidemia and CAD. Further experiments are needed to confirm the relationship and elucidate the mechanism.

## Data Availability Statement

The datasets presented in this study can be found in online repositories. The names of the repository/repositories and accession number(s) can be found in the article/Supplementary Material.

## Author Contributions

TC and ZW conducted statistical analysis and drafted the article. XY contributed to reviewing the article. ZQ and LC edited and revised the article. All authors contributed to manuscript revision, read, and approved the submitted version.

## Funding

This work was supported by the National Natural Science Foundation of China (U2005202), the Fujian Province Major Science and Technology Program (2018YZ001-1), the Natural Science Foundation of Fujian Province (2020J01998 and 2020J02056), and the Fujian Provincial Health Technology Project (2019-ZQN-50).

## Conflict of Interest

The authors declare that the research was conducted in the absence of any commercial or financial relationships that could be construed as a potential conflict of interest.

## Publisher's Note

All claims expressed in this article are solely those of the authors and do not necessarily represent those of their affiliated organizations, or those of the publisher, the editors and the reviewers. Any product that may be evaluated in this article, or claim that may be made by its manufacturer, is not guaranteed or endorsed by the publisher.
