# Supplementary material for: PSRC1 May Affect Coronary Artery Disease Risk by Altering CELSR2, PSRC1, and SORT1 Gene Expression and Circulating Granulin and Apolipoprotein B Protein Levels
Source: Front Cardiovasc Med. 2022 Feb 18;9:763015. doi: 10.3389/fcvm.2022.763015 (PMC8896401; doi:10.3389/fcvm.2022.763015)
Supplement: Supplementary file 1 [file Data_Sheet_1.PDF]

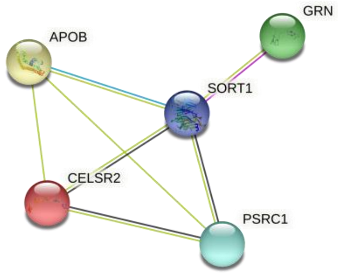

Supplementary Figure S1 The connections between PSRC1, APOB, CELSR2, SORT1 and GRN according to the STRING database.

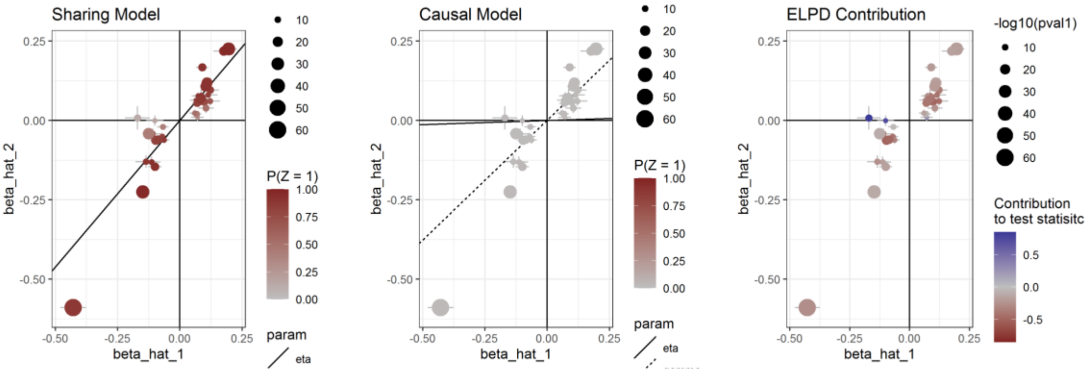

	model1	model2	delta_elpd	se_delta_elpd	z	p
1	null	sharing	-32.0	6.6	-4.8	8.5e-07
2	null	causal	-37.0	8.2	-4.5	3.0e-06
3	sharing	causal	-5.5	2.3	-2.4	7.8e-03

model	gamma	eta	q
Sharing	NA	0.92 (0.71, 1.16)	0.56 (0.37, 0.74)
Causal	0.76 (0.56, 0.95)	0.02 (-1.26, 1.33)	0.05 (0, 0.28)

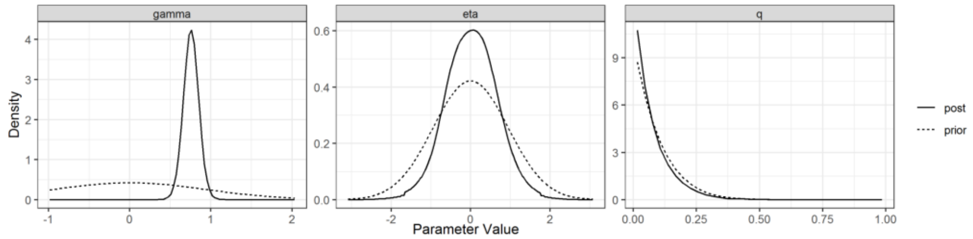

Supplementary Figure S2 Effect-size estimates and variant-level contribution to CAUSE test statistics for circulating apolipoprotein B and LDL-C.

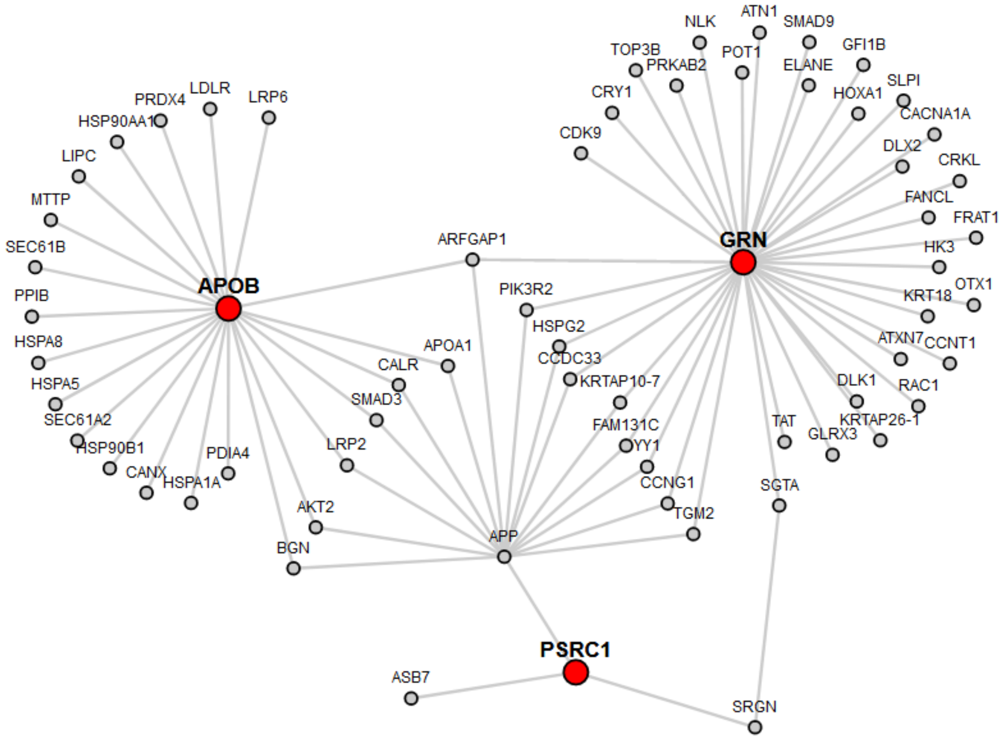

Supplementary Figure S3 The connections between PSRC1, APOB and GRN according to the LENS database.
