# Supplementary material for: PSRC1 May Affect Coronary Artery Disease Risk by Altering CELSR2, PSRC1, and SORT1 Gene Expression and Circulating Granulin and Apolipoprotein B Protein Levels
Source: Front Cardiovasc Med. 2022 Feb 18;9:763015. doi: 10.3389/fcvm.2022.763015 (PMC8896401; doi:10.3389/fcvm.2022.763015)
Supplement: Supplementary file 2 [file Data_Sheet_2.PDF]

Supplementary Table S1 The mRNAs associated with CAD in liver

Gene	CHR	topSNP	topSNP_bp	EA	OA	EAF	b_GWAS	se_GWAS	p_GWAS	b_eQTL	se_eQTL	p_eQTL	Tissue	probeID	b_SMR	se_SMR	p_SMR	p_HEIDI
CELSR2	1	rs12740374	109817590	T	G	0.2989	-0.1135	0.0115	4.63E-23	0.9393	0.0945	2.71E-23	Liver	ENSG00000143126.7	-0.1209	0.0172	2.35E-12	8.48E-01
PSRC1	1	rs12740374	109817590	T	G	0.2989	-0.1135	0.0115	4.63E-23	1.1666	0.1030	1.02E-29	Liver	ENSG00000134222.12	-0.0973	0.0131	9.46E-14	3.68E-02
SORT1	1	rs12740374	109817590	T	G	0.2989	-0.1135	0.0115	4.63E-23	1.1340	0.0909	1.11E-35	Liver	ENSG00000134243.7	-0.1001	0.0129	9.32E-15	3.32E-02
CTSK	1	rs4970926	150673684	C	T	0.4831	0.0382	0.0093	3.99E-05	-0.3398	0.0616	3.38E-08	Liver	ENSG00000143387.8	-0.1123	0.0341	9.82E-04	6.51E-01
TDRD10	1	rs10047079	154468135	C	T	0.2528	0.0388	0.0113	5.95E-04	-0.4189	0.0662	2.48E-10	Liver	ENSG00000163239.8	-0.0927	0.0307	2.54E-03	8.61E-01
RBM6	3	rs2681781	49898273	G	A	0.5278	0.0353	0.0096	2.56E-04	-0.4573	0.0577	2.23E-15	Liver	ENSG00000004534.10	-0.0771	0.0232	8.99E-04	4.26E-02
SNHG18	5	rs431288	9549459	G	A	0.2135	-0.0402	0.0121	8.80E-04	0.8345	0.1032	6.27E-16	Liver	ENSG00000250786.1	-0.0482	0.0157	2.10E-03	1.88E-02
STAT2	12	rs11171806	56733531	A	G	0.1056	-0.0793	0.0207	1.28E-04	0.9499	0.1361	3.01E-12	Liver	ENSG00000170581.9	-0.0835	0.0249	7.85E-04	2.76E-02
SNORA16	13	rs9595617	33057345	T	G	0.3111	-0.0445	0.0101	1.15E-05	0.6204	0.1082	9.91E-09	Liver	ENSG00000212293.1	-0.0717	0.0206	4.95E-04	5.68E-01
PHF11	13	rs1812	50063511	A	G	0.7222	-0.0396	0.0098	5.27E-05	-0.4696	0.0767	9.29E-10	Liver	ENSG00000136147.12	0.0844	0.0250	7.41E-04	4.36E-01
LRRC37A2	17	rs8072451	43893716	T	C	0.1778	0.0468	0.0135	5.22E-04	0.8868	0.0989	2.95E-19	Liver	ENSG00000238083.3	0.0527	0.0163	1.21E-03	2.55E-01
GOSR2	17	rs9911967	45026911	T	C	0.3056	0.0333	0.0093	3.59E-04	0.2724	0.0393	4.39E-12	Liver	ENSG00000108433.11	0.1222	0.0385	1.51E-03	8.66E-01
MXRA7	17	rs2286590	74683666	A	G	0.4389	0.0317	0.0095	8.03E-04	-0.5005	0.0715	2.49E-12	Liver	ENSG00000182534.9	-0.0634	0.0210	2.50E-03	2.35E-02
ZNF100	19	rs6511291	21950402	C	T	0.5389	0.0326	0.0095	6.07E-04	-0.8548	0.0921	1.73E-20	Liver	ENSG00000197020.6	-0.0382	0.0119	1.30E-03	2.88E-02
TGFB1	19	rs15052	41813375	C	T	0.1573	0.0765	0.0148	2.21E-07	0.5254	0.0832	2.74E-10	Liver	ENSG00000105329.5	0.1456	0.0364	6.21E-05	NA
SUSD2	22	rs5760233	24564648	T	G	0.0667	-0.0728	0.0178	4.57E-05	0.5581	0.0972	9.25E-09	Liver	ENSG00000099994.10	-0.1304	0.0392	8.86E-04	7.83E-01

Supplementary Table S2 The mRNAs associated with LDL-C in liver

Gene	CHR	topSNP	topSNP_bp	EA	OA	EAF	b_GWAS	se_GWAS	p_GWAS	b_eQTL	se_eQTL	p_eQTL	Tissue	probeID	b_SMR	se_SMR	p_SMR	p_HEIDI
RHD	1	rs909832	25754025	G	C	0.3944	0.0238	0.0039	8.17E-09	-0.9143	0.1085	3.45E-17	Liver	ENSG00000187010.14	-0.0260	0.0053	7.68E-07	6.51E-04
RHCE	1	rs9689	25688276	G	A	0.5000	0.0328	0.0051	3.63E-09	0.6374	0.0985	9.76E-11	Liver	ENSG00000188672.12	0.0515	0.0113	5.08E-06	8.17E-02
ANGPTL3	1	rs11208007	63176083	T	A	0.3312	-0.0448	0.0039	4.54E-27	-0.3149	0.0616	3.25E-07	Liver	ENSG00000132855.4	0.1423	0.0305	3.05E-06	1.79E-01
CELSR2	1	rs12740374	109817590	T	G	0.2989	-0.1610	0.0044	2.41E-272	0.9393	0.0945	2.71E-23	Liver	ENSG00000143126.7	-0.1714	0.0179	8.39E-22	6.12E-02
PSRC1	1	rs12740374	109817590	T	G	0.2989	-0.1610	0.0044	2.41E-272	1.1666	0.1030	1.02E-29	Liver	ENSG00000134222.12	-0.1380	0.0128	2.88E-27	1.48E-01
SORT1	1	rs12740374	109817590	T	G	0.2989	-0.1610	0.0044	2.41E-272	1.1340	0.0909	1.11E-35	Liver	ENSG00000134243.7	-0.1420	0.0120	3.80E-32	9.62E-03
SYPL2	1	rs4970766	110013955	T	C	0.6778	0.0307	0.0039	4.72E-13	-0.9175	0.0899	1.91E-24	Liver	ENSG00000143028.7	-0.0335	0.0054	4.59E-10	7.39E-05
ATXN7L2	1	rs4970766	110013955	T	C	0.6778	0.0307	0.0039	4.72E-13	-0.6020	0.0806	8.00E-14	Liver	ENSG00000162650.11	-0.0510	0.0094	6.00E-08	1.58E-03
DNAH11	7	rs7809080	21605482	G	C	0.1889	0.0378	0.0062	6.47E-09	0.9553	0.1215	3.83E-15	Liver	ENSG00000105877.13	0.0396	0.0082	1.45E-06	2.33E-01
FADS3	11	rs174578	61605499	A	T	0.3678	-0.0505	0.0046	1.03E-26	-0.4815	0.0923	1.80E-07	Liver	ENSG00000221968.4	0.1049	0.0223	2.44E-06	2.35E-01
ST3GAL4	11	rs7951028	126238394	A	G	0.1389	0.0613	0.0077	5.91E-14	0.7049	0.1161	1.28E-09	Liver	ENSG00000110080.14	0.0870	0.0180	1.39E-06	6.85E-02
NYNRIN	14	rs6573778	24872209	C	T	0.5244	-0.0301	0.0040	3.33E-14	-0.4481	0.0726	6.76E-10	Liver	ENSG00000205978.5	0.0672	0.0141	1.82E-06	1.00E-01
CETP	16	rs17231506	56994528	T	C	0.3636	-0.0572	0.0048	1.33E-28	-0.3151	0.0638	7.73E-07	Liver	ENSG00000087237.6	0.1815	0.0398	4.99E-06	NA
EFCAB13	17	rs6504872	45438952	T	C	0.5000	0.0274	0.0037	3.48E-13	0.6111	0.0767	1.64E-15	Liver	ENSG00000178852.11	0.0448	0.0083	5.83E-08	8.05E-01
SPTLC3	20	rs680379	12969400	G	A	0.5611	0.0243	0.0038	7.96E-10	-0.4483	0.0536	5.84E-17	Liver	ENSG00000172296.8	-0.0542	0.0107	3.75E-07	3.81E-01

Supplementary table s5 Significant pQTLs in the identified genes

SNP_ID	Gene	CHR	Position	Effect allele	Protein gene	Beta	P value pQTL	Study
rs55882046	*PSRC1*	1	109819981	T	C1QTNF1	-0.4979	2.03E-32	Sun B et al. *Nature* 2018
rs55882046	*PSRC1*	1	109819981	T	NEO1	0.2132	6.32E-07	Sun B et al. *Nature* 2018
rs55882046	*PSRC1*	1	109819981	T	HFE2	-0.1946	5.45E-06	Sun B et al. *Nature* 2018
rs55882046	*PSRC1*	1	109819981	T	CA10	-0.238	2.49E-08	Sun B et al. *Nature* 2018
rs55882046	*PSRC1*	1	109819981	T	PLA2G12B	-0.3381	1.79E-15	Sun B et al. *Nature* 2018
rs55882046	*PSRC1*	1	109819981	T	GRN	-0.6518	7.56E-56	Sun B et al. *Nature* 2018
rs6677122	*PSRC1*	1	109820110	T	FJX1	-0.283	5.19E-06	Sun B et al. *Nature* 2018
rs6677122	*PSRC1*	1	109820110	T	CA10	-0.3343	6.97E-08	Sun B et al. *Nature* 2018
rs6677122	*PSRC1*	1	109820110	T	C1QTNF1	-0.4363	1.67E-12	Sun B et al. *Nature* 2018
rs6677122	*PSRC1*	1	109820110	T	GRN	-0.6108	2.19E-23	Sun B et al. *Nature* 2018
rs11577931	*PSRC1*	1	109820884	A	GRN	0.6582	2.19E-56	Sun B et al. *Nature* 2018
rs11577931	*PSRC1*	1	109820884	A	HFE2	0.1968	4.97E-06	Sun B et al. *Nature* 2018
rs11577931	*PSRC1*	1	109820884	A	PLA2G12B	0.3439	9.35E-16	Sun B et al. *Nature* 2018
rs11577931	*PSRC1*	1	109820884	A	CA10	0.2466	9.76E-09	Sun B et al. *Nature* 2018
rs11577931	*PSRC1*	1	109820884	A	C1QTNF1	0.5013	2.13E-32	Sun B et al. *Nature* 2018
rs11577931	*PSRC1*	1	109820884	A	NEO1	-0.2101	1.03E-06	Sun B et al. *Nature* 2018
rs17035949	*PSRC1*	1	109820919	T	GRN	0.6156	7.11E-24	Sun B et al. *Nature* 2018
rs17035949	*PSRC1*	1	109820919	T	PTH2	0.2806	5.61E-06	Sun B et al. *Nature* 2018
rs17035949	*PSRC1*	1	109820919	T	CA10	0.3229	1.74E-07	Sun B et al. *Nature* 2018
rs17035949	*PSRC1*	1	109820919	T	C1QTNF1	0.447	3.64E-13	Sun B et al. *Nature* 2018
rs17035949	*PSRC1*	1	109820919	T	FJX1	0.2864	3.58E-06	Sun B et al. *Nature* 2018
rs583104	*PSRC1*	1	109821307	T	SLC5A8	0.1598	9.07E-08	Sun B et al. *Nature* 2018
rs583104	*PSRC1*	1	109821307	T	APOB	0.1379	3.99E-06	Sun B et al. *Nature* 2018
rs583104	*PSRC1*	1	109821307	T	FJX1	0.2562	6.34E-18	Sun B et al. *Nature* 2018
rs583104	*PSRC1*	1	109821307	T	PLA2G12B	0.4212	1.76E-47	Sun B et al. *Nature* 2018
rs583104	*PSRC1*	1	109821307	T	GRN	0.8126	1.71E-206	Sun B et al. *Nature* 2018
rs583104	*PSRC1*	1	109821307	T	NEO1	-0.2245	4.94E-14	Sun B et al. *Nature* 2018
rs583104	*PSRC1*	1	109821307	T	STC1	0.1438	1.51E-06	Sun B et al. *Nature* 2018
rs583104	*PSRC1*	1	109821307	T	SELE	0.136	5.40E-06	Sun B et al. *Nature* 2018
rs583104	*PSRC1*	1	109821307	T	LRP1B	0.1482	7.18E-07	Sun B et al. *Nature* 2018
rs583104	*PSRC1*	1	109821307	T	CA10	0.2869	3.24E-22	Sun B et al. *Nature* 2018
rs583104	*PSRC1*	1	109821307	T	C1QTNF1	0.6339	2.81E-114	Sun B et al. *Nature* 2018
rs583104	*PSRC1*	1	109821307	T	HFE2	0.2152	5.14E-13	Sun B et al. *Nature* 2018
rs602633	*PSRC1*	1	109821511	T	STC1	-0.1552	3.02E-07	Sun B et al. *Nature* 2018
rs602633	*PSRC1*	1	109821511	T	APOB	-0.1372	5.95E-06	Sun B et al. *Nature* 2018
rs602633	*PSRC1*	1	109821511	T	NEO1	0.2167	7.21E-13	Sun B et al. *Nature* 2018
rs602633	*PSRC1*	1	109821511	T	C1QTNF1	-0.6371	3.14E-112	Sun B et al. *Nature* 2018
rs602633	*PSRC1*	1	109821511	T	PLA2G12B	-0.4144	7.99E-45	Sun B et al. *Nature* 2018
rs602633	*PSRC1*	1	109821511	T	SLC5A8	-0.1643	5.88E-08	Sun B et al. *Nature* 2018
rs602633	*PSRC1*	1	109821511	T	SELE	-0.1374	5.77E-06	Sun B et al. *Nature* 2018
rs602633	*PSRC1*	1	109821511	T	CA10	-0.2928	1.67E-22	Sun B et al. *Nature* 2018
rs602633	*PSRC1*	1	109821511	T	FJX1	-0.2596	6.43E-18	Sun B et al. *Nature* 2018
rs602633	*PSRC1*	1	109821511	T	HFE2	-0.2048	1.19E-11	Sun B et al. *Nature* 2018
rs602633	*PSRC1*	1	109821511	T	GRN	-0.8143	2.73E-201	Sun B et al. *Nature* 2018
rs602633	*PSRC1*	1	109821511	T	LRP1B	-0.1508	6.46E-07	Sun B et al. *Nature* 2018
rs4970835	*PSRC1*	1	109821588	A	C1QTNF1	0.2	4.74E-11	Sun B et al. *Nature* 2018
rs4970835	*PSRC1*	1	109821588	A	GRN	0.2478	2.88E-16	Sun B et al. *Nature* 2018
rs4970836	*PSRC1*	1	109821797	A	GRN	0.8048	4.14E-204	Sun B et al. *Nature* 2018
rs4970836	*PSRC1*	1	109821797	A	PLA2G12B	0.4224	4.65E-48	Sun B et al. *Nature* 2018

rs4970836	*PSRC1*	1	109821797	A	LRP1B	0.1482	6.59E-07	Sun B et al. *Nature* 2018
rs4970836	*PSRC1*	1	109821797	A	CA10	0.285	4.42E-22	Sun B et al. *Nature* 2018
rs4970836	*PSRC1*	1	109821797	A	NEO1	-0.2236	4.22E-14	Sun B et al. *Nature* 2018
rs4970836	*PSRC1*	1	109821797	A	FJX1	0.2585	2.48E-18	Sun B et al. *Nature* 2018
rs4970836	*PSRC1*	1	109821797	A	STC1	0.1349	5.99E-06	Sun B et al. *Nature* 2018
rs4970836	*PSRC1*	1	109821797	A	APOB	0.1398	2.72E-06	Sun B et al. *Nature* 2018
rs4970836	*PSRC1*	1	109821797	A	SLC5A8	0.1591	9.35E-08	Sun B et al. *Nature* 2018
rs4970836	*PSRC1*	1	109821797	A	C1QTNF1	0.6268	1.45E-112	Sun B et al. *Nature* 2018
rs4970836	*PSRC1*	1	109821797	A	HFE2	0.2177	1.91E-13	Sun B et al. *Nature* 2018
rs4970837	*PSRC1*	1	109822008	T	FJX1	0.1766	1.32E-11	Sun B et al. *Nature* 2018
rs4970837	*PSRC1*	1	109822008	T	NEO1	-0.1823	2.86E-12	Sun B et al. *Nature* 2018
rs4970837	*PSRC1*	1	109822008	T	HFE2	0.1462	2.40E-08	Sun B et al. *Nature* 2018
rs4970837	*PSRC1*	1	109822008	T	PLA2G12B	0.292	1.07E-29	Sun B et al. *Nature* 2018
rs4970837	*PSRC1*	1	109822008	T	GRN	0.5527	1.09E-112	Sun B et al. *Nature* 2018
rs4970837	*PSRC1*	1	109822008	T	CA10	0.188	5.89E-13	Sun B et al. *Nature* 2018
rs4970837	*PSRC1*	1	109822008	T	C1QTNF1	0.4345	1.28E-66	Sun B et al. *Nature* 2018
rs1277930	*PSRC1*	1	109822143	A	NEO1	-0.2253	2.71E-14	Sun B et al. *Nature* 2018
rs1277930	*PSRC1*	1	109822143	A	LRP1B	0.1496	5.16E-07	Sun B et al. *Nature* 2018
rs1277930	*PSRC1*	1	109822143	A	FJX1	0.2551	5.27E-18	Sun B et al. *Nature* 2018
rs1277930	*PSRC1*	1	109822143	A	STC1	0.1353	5.62E-06	Sun B et al. *Nature* 2018
rs1277930	*PSRC1*	1	109822143	A	C1QTNF1	0.6248	7.32E-112	Sun B et al. *Nature* 2018
rs1277930	*PSRC1*	1	109822143	A	CA10	0.2824	1.04E-21	Sun B et al. *Nature* 2018
rs1277930	*PSRC1*	1	109822143	A	HFE2	0.2168	2.40E-13	Sun B et al. *Nature* 2018
rs1277930	*PSRC1*	1	109822143	A	SLC5A8	0.16	7.16E-08	Sun B et al. *Nature* 2018
rs1277930	*PSRC1*	1	109822143	A	APOB	0.1391	3.05E-06	Sun B et al. *Nature* 2018
rs1277930	*PSRC1*	1	109822143	A	GRN	0.8017	1.48E-202	Sun B et al. *Nature* 2018
rs1277930	*PSRC1*	1	109822143	A	PLA2G12B	0.4199	1.64E-47	Sun B et al. *Nature* 2018
rs599839	*PSRC1*	1	109822166	G	GRN	-0.7453	3.32E-51	Suhre K et al. *Nat Commun* 2017
rs599839	*PSRC1*	1	109822166	G	CAT	-0.329	3.34E-11	Suhre K et al. *Nat Commun* 2017
rs599839	*PSRC1*	1	109822166	G	RGMA	-0.3145	1.32E-09	Suhre K et al. *Nat Commun* 2017
rs599839	*PSRC1*	1	109822166	A	FJX1	0.2569	6.65E-18	Sun B et al. *Nature* 2018
rs599839	*PSRC1*	1	109822166	A	LRP1B	0.1491	6.70E-07	Sun B et al. *Nature* 2018
rs599839	*PSRC1*	1	109822166	A	STC1	0.1371	4.88E-06	Sun B et al. *Nature* 2018
rs599839	*PSRC1*	1	109822166	A	GRN	0.8071	1.00E-200	Sun B et al. *Nature* 2018
rs599839	*PSRC1*	1	109822166	A	APOB	0.1389	3.66E-06	Sun B et al. *Nature* 2018
rs599839	*PSRC1*	1	109822166	A	C1QTNF1	0.6293	4.40E-111	Sun B et al. *Nature* 2018
rs599839	*PSRC1*	1	109822166	A	PLA2G12B	0.421	4.00E-47	Sun B et al. *Nature* 2018
rs599839	*PSRC1*	1	109822166	A	CA10	0.2815	2.59E-21	Sun B et al. *Nature* 2018
rs599839	*PSRC1*	1	109822166	A	SLC5A8	0.1607	8.48E-08	Sun B et al. *Nature* 2018
rs599839	*PSRC1*	1	109822166	A	NEO1	-0.2291	1.83E-14	Sun B et al. *Nature* 2018
rs599839	*PSRC1*	1	109822166	A	HFE2	0.2195	2.12E-13	Sun B et al. *Nature* 2018
rs72703210	*PSRC1*	1	109823962	T	GRN	0.353	6.78E-09	Sun B et al. *Nature* 2018
rs72703210	*PSRC1*	1	109823962	T	PLA2G12B	0.3105	3.42E-07	Sun B et al. *Nature* 2018
rs35358959	*PSRC1*	1	109824250	A	GRN	-0.5654	7.92E-37	Sun B et al. *Nature* 2018
rs35358959	*PSRC1*	1	109824250	A	C1QTNF1	-0.4528	8.12E-24	Sun B et al. *Nature* 2018
rs35358959	*PSRC1*	1	109824250	A	PLA2G12B	-0.3578	2.45E-15	Sun B et al. *Nature* 2018
rs138361368	*PSRC1*	1	109825558	A	C1QTNF1	-0.5441	5.26E-10	Sun B et al. *Nature* 2018
rs138361368	*PSRC1*	1	109825558	A	GRN	-0.6325	4.59E-13	Sun B et al. *Nature* 2018
rs657420	*PSRC1*	1	109826136	T	GRN	0.2228	1.34E-19	Sun B et al. *Nature* 2018
rs657420	*PSRC1*	1	109826136	T	C1QTNF1	0.1514	8.81E-10	Sun B et al. *Nature* 2018
rs657420	*PSRC1*	1	109826136	T	PLA2G12B	0.1473	2.47E-09	Sun B et al. *Nature* 2018

rs68104325	*PSRC1*	1	109826760	T	GRN	0.4743	1.13E-47	Sun B et al. *Nature* 2018
rs68104325	*PSRC1*	1	109826760	T	CA10	0.1653	8.67E-07	Sun B et al. *Nature* 2018
rs68104325	*PSRC1*	1	109826760	T	PLA2G12B	0.2883	6.04E-18	Sun B et al. *Nature* 2018
rs68104325	*PSRC1*	1	109826760	T	C1QTNF1	0.3658	2.16E-28	Sun B et al. *Nature* 2018
rs672569	*PSRC1*	1	109827253	A	PLA2G12B	-0.2811	2.02E-17	Sun B et al. *Nature* 2018
rs672569	*PSRC1*	1	109827253	A	C1QTNF1	-0.3566	2.25E-27	Sun B et al. *Nature* 2018
rs672569	*PSRC1*	1	109827253	A	CA10	-0.1572	2.52E-06	Sun B et al. *Nature* 2018
rs672569	*PSRC1*	1	109827253	A	GRN	-0.4725	6.91E-48	Sun B et al. *Nature* 2018
rs146292694	*PSRC1*	1	109828401	T	GRN	-0.1999	3.37E-16	Sun B et al. *Nature* 2018
rs146292694	*PSRC1*	1	109828401	T	C1QTNF1	-0.1293	1.47E-07	Sun B et al. *Nature* 2018
rs146292694	*PSRC1*	1	109828401	T	PLA2G12B	-0.1202	1.14E-06	Sun B et al. *Nature* 2018
rs595770	*PSRC1*	1	109829590	T	PLA2G12B	-0.1156	2.87E-06	Sun B et al. *Nature* 2018
rs595770	*PSRC1*	1	109829590	T	GRN	-0.1635	3.01E-11	Sun B et al. *Nature* 2018
rs141844707	*PSRC1*	1	109830993	T	C1QTNF1	-0.4217	3.19E-22	Sun B et al. *Nature* 2018
rs141844707	*PSRC1*	1	109830993	T	PLA2G12B	-0.3218	2.03E-13	Sun B et al. *Nature* 2018
rs141844707	*PSRC1*	1	109830993	T	GRN	-0.5391	6.74E-36	Sun B et al. *Nature* 2018
